# Network Analysis Implicates Alpha-Synuclein (*Snca*) in the Regulation of Ovariectomy-Induced Bone Loss

**DOI:** 10.1038/srep29475

**Published:** 2016-07-05

**Authors:** Gina Calabrese, Larry D. Mesner, Patricia L. Foley, Clifford J. Rosen, Charles R. Farber

**Affiliations:** 1 Center for Public Health Genomics, University of Virginia, Charlottesville, Virginia 22908, USA; 2 Department of Microbiology and Immunology, Georgetown University, Washington, DC 20007, USA; 3 Maine Medical Center Research Institute, 81 Research Drive, Scarborough, Maine 04074, USA; 4 Departments of Public Health Science and Biochemistry and Molecular Genetics, University of Virginia, Charlottesville, Virginia 22908, USA

## Abstract

The postmenopausal period in women is associated with decreased circulating estrogen levels, which accelerate bone loss and increase the risk of fracture. Here, we gained novel insight into the molecular mechanisms mediating bone loss in ovariectomized (OVX) mice, a model of human menopause, using co-expression network analysis. Specifically, we generated a co-expression network consisting of 53 gene modules using expression profiles from intact and OVX mice from a panel of inbred strains. The expression of four modules was altered by OVX, including module 23 whose expression was decreased by OVX across all strains. Module 23 was enriched for genes involved in the response to oxidative stress, a process known to be involved in OVX-induced bone loss. Additionally, module 23 homologs were co-expressed in human bone marrow. Alpha synuclein (*Snca*) was one of the most highly connected “hub” genes in module 23. We characterized mice deficient in *Snca* and observed a 40% reduction in OVX-induced bone loss. Furthermore, protection was associated with the altered expression of specific network modules, including module 23. In summary, the results of this study suggest that *Snca* regulates bone network homeostasis and ovariectomy-induced bone loss.

Osteoporosis is a condition characterized by low bone mineral density (BMD) and an increased risk of fracture^1^. Fractures are a major burden to the U.S. healthcare system, both in terms of cost (~$18 billion) and resource utilization^2^. Additionally, of the ~300,000 people over the age of 50 that suffer a hip fracture each year, 20% will die in the subsequent 12 months and 50% of the survivors will not return to their prior independent living status^3^.

Osteoporosis primarily affects postmenopausal women due to decreased estrogen levels^4^, which accelerate bone loss. Estrogen is a regulator of bone-forming osteoblasts and bone-resorbing osteoclasts^5^. In states of estrogen deficiency, both bone formation and resorption are increased; however, resorption outpaces formation resulting in lower BMD and higher rates of fracture. To make this situation worse, women experiencing the most rapid postmenopausal bone loss are at an even higher risk for fracture independent of BMD^6^. As a result, postmenopausal bone loss is the most significant contributor to poor skeletal health in aging women.

Cellular networks assimilate, organize and transmit genetic and environmental information and in doing so determine the cellular response to perturbations, such as estrogen deficiency^7^. Many groups have demonstrated that reconstructing cellular networks is an effective approach to identify novel pathways and genes that participate in specific disease-related processes^8^,^9^,^10^,^11^. One of the most popular network reconstruction methods is Weighted Gene Co-expression Network Analysis (WGCNA). WGCNA quantifies correlational relationships among genes on a genome-wide scale using global expression data collected across multiple perturbations^12^,^13^. WGCNA networks are modular, with distinct modules representing dense clusters of genes that are highly co-expressed. Modules are often enriched for genes that are members of the same or similar pathways^13^ and pathways can be linked to a particular disease by identifying modules whose behavior correlates with a disease-related phenotype^14^,^15^,^16^,^17^,^18^. Furthermore, the most important genes in a module can be identified by focusing on “hubs”, which are the genes most strongly connected (or correlated) with the largest number of other module genes^13^. Studies have found that intramodular connectivity correlates with biologically relevant properties^13^. For example, we have demonstrated that hub genes in a module associated with BMD in humans were more likely to be genetically associated with BMD than non-hub genes^14^. We have also shown that hubs in mouse chondrocyte- and osteoblast-associated modules play key roles in the differentiation of these two cell-types as well as BMD^15^,^16^.

In the current study, we used a mouse model of postmenopausal bone loss (ovariectomy (OVX)-induced bone loss) and variation in gene expression generated by the divergent genetic backgrounds of inbred mouse strains to construct a bone co-expression network in intact and ovariectomized mice. In our analysis, we identified a module of genes whose expression is associated with OVX-induced bone loss. Furthermore, we demonstrated that the hub gene of this module, alpha-synuclein (*Snca*), is a key mediator of the expression of specific network modules and the skeletal response to estrogen deficiency.

## Results

### Effects of OVX on the bone transcriptome

We measured OVX-induced bone loss at the spine (L4 vertebrae) in three inbred strains (BALB/cJ, C3H/HeJ and C57BL6/J) of mice, four weeks post-surgery. Our goal was to maximize variation in phenotypic and transcriptional responses. Therefore, strains were chosen based on prior studies^19^,^20^,^21^,^22^ demonstrating significant differences in baseline bone mass and magnitude of trabecular bone loss in response to OVX. As previously reported^19^,^20^,^21^,^22^, we observed significant (P < 0.05) effects of strain genotype and surgery (OVX vs. SHAM) and suggestive (P < 0.10) genotype x surgery interactions on trabecular bone parameters (Table 1).

To identify genes differentially expressed due to OVX, we profiled L5 vertebrae from all six groups (three strains by two surgical groups; N = 4/strain/surgery group). In total, we identified 5,302 probes (4,463 unique genes) as differentially expressed (FDR ≤ 0.10) by strain (Supplementary Table 1). In contrast, 168 probes (153 unique genes) were differentially expressed (FDR ≤ 0.10) as a function of OVX (Supplementary Table 2). No genes demonstrated significant strain by surgery interactions. The group of genes differentially expressed due to OVX was enriched for transcripts involved “immune system development” (FDR = 1.0 × 10^−5^) and “leukocyte/B-cell activation” (FDR = 1.3 × 10^−3^). These data indicate that estrogen deficiency elicits subtle transcriptional effects predominantly affecting leukocyte expression. Furthermore, based on these data alone, it was unclear which genes were “drivers” of bone loss.

### Reconstruction of a bone co-expression network and identification of modules influenced by OVX

**Figure 1:**
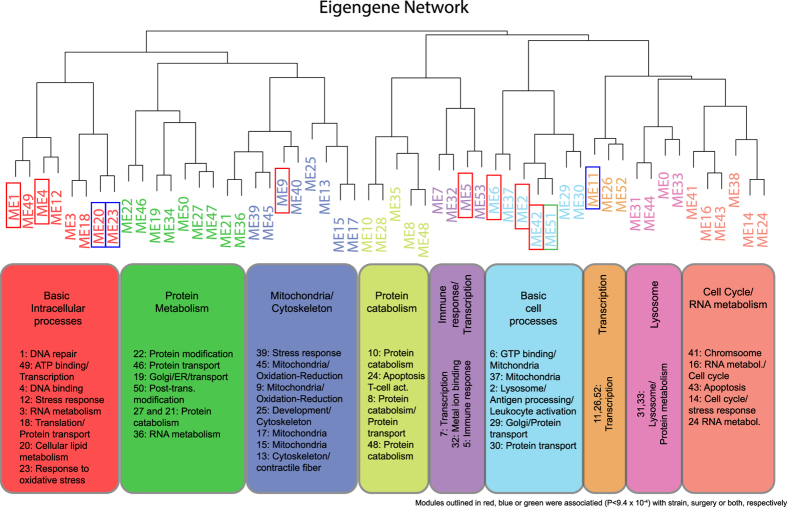
Organization of the bone co-expression network based on module eigengenes. An eigengene network was constructed by hierarchical clustering of the eigengenes for all 53 modules in the bone network. Modules are colored coded based on a general similarity in gene ontology enrichments. Modules outlined in red, blue or green were associated (P < 9.4 × 10^−3^) with strain, surgery or both, respectively.

To generate a systems-level view of the L5 expression profiles, we constructed a co-expression network using WGCNA. The constructed network consisted of 11,738 probes (representing 8,947 unique genes) partitioned into 53 co-expression modules ranging in size from 26 to 1124 probes (Fig. 1 and Supplemental Table 3). All modules were enriched for a wide range of basic and specific cellular functions (Fig. 1 and Supplemental Table 4). Modules whose eigengenes clustered together were typically enriched for the same gene ontology (GO) terms (Fig. 1).

We tested the effects of strain, surgery and strain by surgery interactions on each module eigengene (a vector of values summarizing the overall behavior of each module) to identify modules of potential biological interest to bone loss. Eight modules (modules 1, 2, 4, 5, 6, 9, 42 and 51) were significantly (P < 9.4 × 10^−4^, Bonferroni corrected for 53 tested modules) influenced by strain and four (modules 11, 20, 23 and 51) were influenced by OVX (Table 2).

To identify genes potentially regulating OVX-induced bone loss, we focused our attention on the four modules differentially expressed as a function of OVX, independent of strain (Table 2). The four modules contained a total of 692 probes (representing 644 genes), 4.5 times more genes than identified by the standard differential expression analysis. The eigengenes for modules 20, 23 and 51 were decreased by OVX, whereas, the module 11 eigengene was increased by OVX. A GO enrichment analysis of the four modules revealed that modules 11, 20 and 51 were enriched for general processes. The top enrichment terms in these modules were “cellular process” (module 11 FDR = 1.2 × 10^−8^), “intracellular part” (module 20 FDR = 9.0 × 10^−4^) and “fibronectin binding” (module 51 FDR = 0.11) (Supplemental Table 4). In contrast, the top enriched term for module 23 was “cellular response to oxidative stress” (FDR = 0.02) and oxidative stress has been associated with increased OVX-induced bone loss^23^. Therefore, while modules 11, 20 and 51 may contain genes important for OVX-induced bone loss, module 23 was more likely to contain genes that were directly “driving” bone loss as a response to OVX. Module 23 contained four genes belonging to the gene category “response to oxidative stress” including alpha-synuclein (*Snca*), peroxiredoxin 2 (*Prdx2*), peroxiredoxin 3 (*Prdx3*) and glutathione peroxidase 1 (*Gpx1*). The last three encode for key antioxidants. In addition, many genes involved in glutathione (a major cellular antioxidant) metabolism were members of module 23, including glutamate-cysteine ligase (*Gclm*), glutaredoxin (*Glrx5*), glutathione S-transferase mu 5 (*Gstm5*) and glutathione S-transferase mu 6 (*Gstm6*). *Snca* was one of the most highly connected genes (based on Kme; defined as a gene’s correlation with its module eigengene) in module 23 (ranked 5^th^ out of 131).

**Figure 2:**
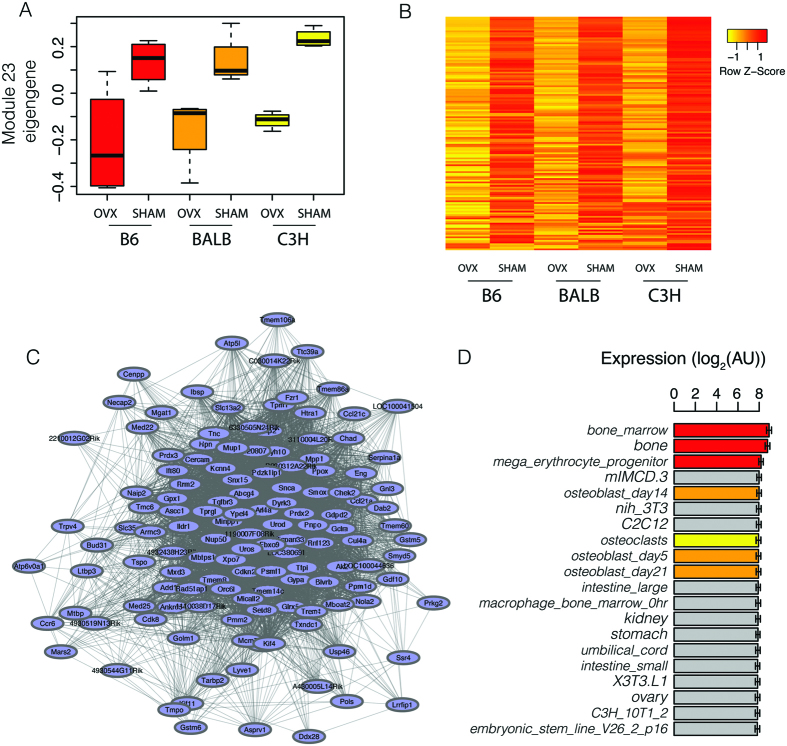
Identification and characterization of module 23. (**A**) Module 23 gene expression levels are decreased by OVX across three inbred strains (N = 4 biological replicates per strain and surgical group). (**B**) Heatmap demonstrating the strong co-expression of module 23 genes. (**C**) The module 23 network where lines connecting genes represent co-expression relationships in the top 50% of all topological overlap measures (TOMs). (**D**) Module 23 genes are highly expressed in bone marrow and bone as compared to 96 other mouse tissue and cell types (the 20 samples with the highest mean module 23 expression are shown). Values in (**D**) are means ± s.e.m. B6 = C57BL6/J, BALB = BALB/cJ, C3H = C3H/HeJ, OVX = ovariectomized, SHAM = sham operated and AU = arbitrary units.

Based on the enrichment for genes involved in the response to oxidative stress, which has been linked to ovariectomy-induced bone loss, we focused on module 23. The expression of module 23 genes (136 array probes representing 131 unique genes) was decreased in OVX mice (Fig. 2A). As one would expect, module 23 genes demonstrated highly similar expression patterns across surgery and strain groups (Fig. 2B). In addition to *Snca*, many of the most highly connected genes in module 23 were highly expressed in erythrocytes and involved in their function, such as uroporphyrinogen III synthase (*Uros*; the most connected), dual-specificity tyrosine-(Y)-phosphorylation regulated kinase 3 (*Dyrk3*), transforming growth factor, beta receptor III (*Tgfbr3*), among many others (Fig. 2C). Consistent with this observation, a broad survey of expression profiles revealed that module 23 genes were nearly 2-fold more highly expressed in bone marrow and bone (plus marrow) than a panel of 96 other mouse cell types and tissues (Fig. 2D). Module 23 genes were also highly expressed in primary osteoblasts, at three different points during differentiation, and primary osteoclasts (these four samples were in the top 10 of the 96 samples) (Fig. 2D).

### Module 23 homologs are co-expressed in human bone marrow

**Figure 3:**
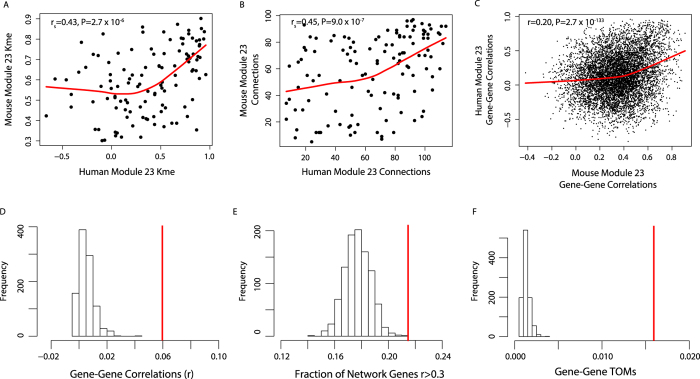
Human module 23 homologs are co-expressed in bone marrow. Shown in (**A–C**) are correlations between connectivity (Kme) (**A**), the number of connections (**B**) and pairwise gene-gene correlations between mouse and human module 23 genes. Shown in **D**–**F** are mean distributions of pairwise gene correlations (**A**), fraction of pairwise correlations >|0.30| (**B**) and pairwise TOMs (**C**) for 1000 randomly selected human homolog sets (N = 123, same size as module 23) compared to the mean of module 23 homologs in human bone marrow (red line). TOM = topological overlap measure (see Methods).

We reasoned that if module 23 represented a truly cohesive and functional gene set then its members would also be co-expressed in humans. To test this prediction, we quantified the co-expression relationships of module 23 homologs in bone marrow from normal human subjects (N = 25). Homologs were identified for 123 of the 131 mouse module 23 genes. We observed strong positive correlations for Kme (r_s_ = 0.43, P = 2.7 × 10^−6^) and the number of connections (r_s_ = 0.45, P = 9.0 × 10^−7^) between mouse and human genes when comparing the upper half of topological overlap measures (TOMs, a measure of how strongly two genes are correlated with the same sets of genes) (Fig. 3A,B). We then compared pairwise gene correlations for the same pairs in both networks and observed a positive correlation (r = 0.20, P = 2.7 × 10^−13 ^3) (Fig. 3C), indicating that genes more highly co-expressed in the mouse were more highly co-expressed in humans. In the human network, we also compared the distribution of pairwise correlations, the percent of pairwise correlations with an r> = 0.30 (which approximates a nominally (P < 0.05) significant correlation for this given sample size) and TOMs for module 23 homologs. When compared to 1000 sets of 123 randomly selected genes these data indicated that module 23 homologs were more highly connected with one another than random gene sets (Fig. 3D–F). These data support the hypothesis that module 23 represents a biologically relevant co-expressed gene set.

### Snca is a hub gene for module 23

**Figure 4:**
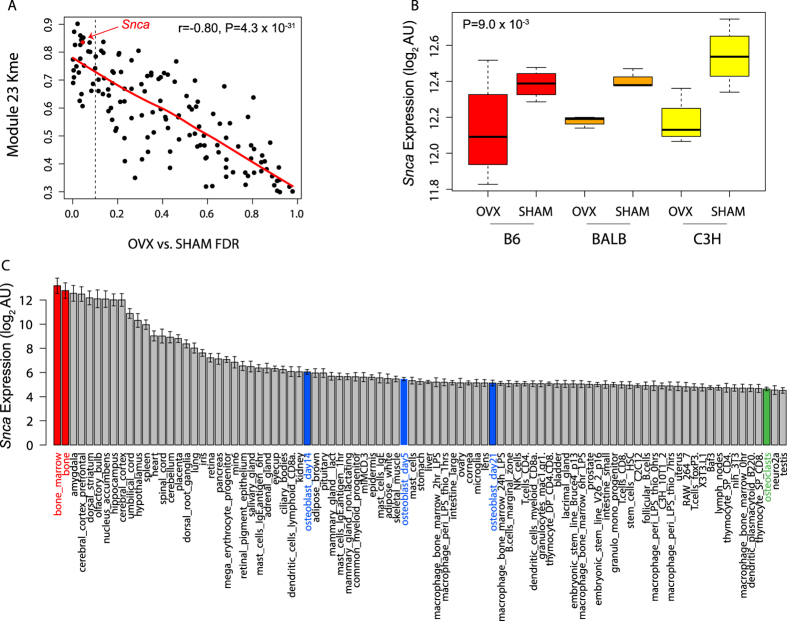
Alpha-synuclein (*Snca*) is a module 23 hub gene. (**A**) We observed a positive correlation (r_s_ = −0.80, P = 4.3 × 10^−31^) between Kme and the magnitude of differential expression between OVX and SHAM (FDR) across the three inbred strains. The diagonal line represents the smoothed best fit. Genes to the left of the vertical dashed line were differentially expressed at an FDR < 10%. (**B**) *Snca* expression is lower in all three inbred strains (P = 9.0 × 10^−3^). (**C**) *Snca* is highly expressed in bone marrow (in red) and expressed in differentiating osteoblasts (in blue).

We next characterized module 23 hub genes as a way to prioritize and identify individual genes impacting OVX-induced bone loss. To identify hubs, we ranked genes based on Kme. Prior studies have found that module hub genes often play key roles in regulating the overall behavior of a module and phenotypes (in the case of modules associated with a phenotype, such as module 23 and OVX). In support of this notion, we found that module 23 genes with higher kme values tended to have lower differential expression FDR values (r = −0.80, P = 4.3 × 10^−31^) (Fig. 4A). *Snca* was among the most connected (5 of 131) and differentially expressed genes in module 23 (Fig. 4A). *Snca* was also the most highly connected gene with a known role in the regulation of oxidative stress^24^. Mutations in *SNCA* have been linked to familial and sporadic cases of Parkinson’s Disease^25^,^26^,^27^. Though its precise molecular function is unclear, it has been shown to promote bone marrow oxidative stress^24^. The expression of *Snca* mirrors that of the module 23 eigengene (Fig. 4B). Of 96 mouse cell lines and tissues, *Snca* is most highly expressed in bone marrow (Fig. 4C), consistent with the overall expression of module 23 genes.

### Snca deficiency protects against OVX-induced bone loss

**Figure 5:**
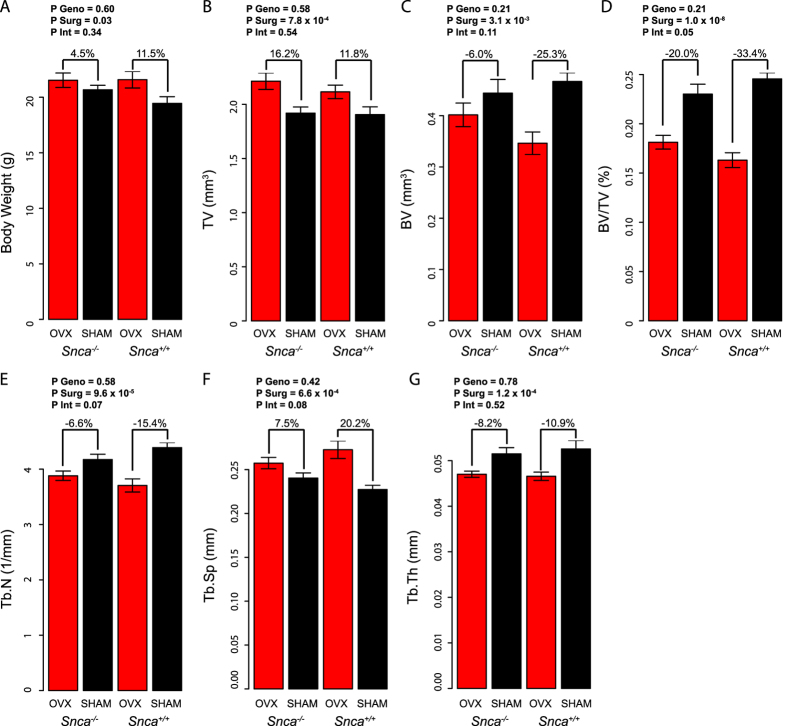
*Snca* deficiency protects mice from OVX-induced bone loss. Body weight (**A**), total volume (TV) (**B**), bone volume (BV) (**C**), bone volume fraction (BV/TV) (**D**), trabecular number (Tb.N) (**E**), trabecular thickness (Tb.Th) (**F**) and trabecular separation (Tb.Sp) of the L4 vertebrae in OVX and SHAM mice from both *Snca* genotypes (*Snca*^−/−^ and *Snca*^+/+^) (N = 10 for both OVX groups, N = 9 for *Snca*^+/+^ SHAM and N = 6 for *Snca*^+/+^ OVX). Percent differences between OVX and SHAM within each genotype are provided. Effect of genotype (*Snca*^−/−^ and *Snca*^+/+^), surgery (OVX and SHAM) and interaction between surgery and genotype determined using ANOVA. P geno = P-value for the effect of genotype, P Surg = P-value for the effect of surgery and P Int = P-value for the effect of the interaction between genotype and surgery. P Int ≤ 0.05 indicates a significant difference due to OVX between genotypes. All values are means ± s.e.m.

Based on its role as a hub in module 23 and the evidence to suggest that it promotes bone marrow oxidative stress^24^, we hypothesized that *Snca* promoted OVX-induced bone loss. To test this hypothesis, we measured L4 trabecular bone mass in *Snca* knockout (*Snca*^−/−^) and wild-type littermates (*Snca*^+/+^) after OVX and SHAM surgeries. OVX increased (P = 0.03) body weight in both genotypes (Fig. 5A). In general, OVX increased (P = 7.8 × 10^−4^) total volume (TV) (Fig. 5B) and decreased (P = 3.1 × 10^−3^) bone volume (BV) (Fig. 5C). However, the magnitude of bone volume fraction (BV/TV) loss due to OVX was significantly (interaction P = 0.05) less in *Snca*^−/−^ mice than littermate controls (Fig. 5D). *Snca*^−/−^ mice lost 20.0% of BV/TV, compared to a 33.4% decrease of BV/TV in *Snca*^+/+^ females (Fig. 5D). This was primarily due to preferential preservation of trabecular number (Tb.N; interaction P = 0.07) and spacing (Tb.Sp; interaction P = 0.08) after OVX in *Snca*^−/−^ mice (Fig. 5E,F). There was no effect of an OVX by genotype interaction on trabecular thickness (Tb.Th; interaction P = 0.52) (Fig. 5G).

### *Snca* deficiency alters the expression of specific bone network modules

As a module 23 hub gene, we hypothesized that *Snca* regulated OVX-induced bone loss by altering the expression of genes in its own module. To test this hypothesis, we profiled the L5 transcriptome in each *Snca* genotype. *Snca* transcripts were undetectable in mutant mice (data not shown). For all other genes, two metrics were generated: percent difference in expression between *Snca* genotypes and the difference in behavior after OVX in each genotype (% difference in WT (wild-type)-% difference in MT (mutant)). We then assigned each gene to their respective L5 module from the *original* multi-strain network and calculated the mean difference for both measures within each of the 53 modules. Modules were deemed perturbed if significant (P < 9.4 × 10^−4^) using Gene Set Enrichment Analysis (GSEA)^28^. To account for noise in the expression data, genes were ranked based on P-values for the differential expression between genotypes or P-values from the ANOVA genotype x surgery interaction terms.

**Figure 6:**
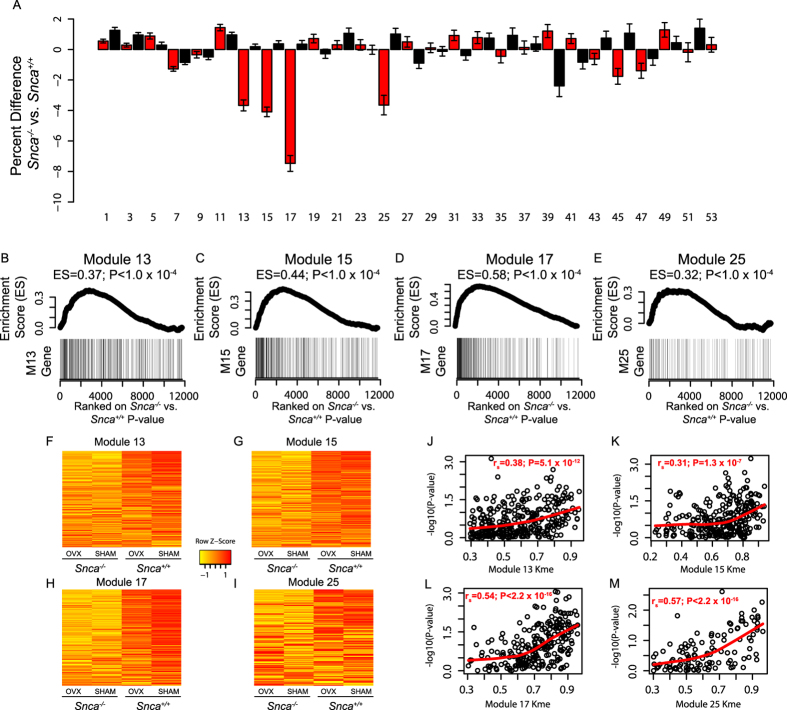
*Snca* deficiency specifically alters network modules 13, 15, 17 and 25. (**A**) The expression of genes in modules 13, 15, 17 and 23 was decreased as a function of *Snca* deficiency. (**B–E**) Gene Set Enrichment Analysis (GSEA) results for the four modules. The differences in expression between genotypes were ranked from most significant to least significant. Genes in all four modules tend to be among the most significant. (**F**–**I**) Genes in all four modules were more lowly expressed in *Snca*^−/−^ mice. (**F**) We observed positive correlations between Kme and differential expression (−log10(Pvalue)) in all four modules. Values in A are means ± s.e.m.

In the comparison between genotypes (*Snca*^−/−^ versus *Snca*^+/+^), genes in four modules were significantly altered (Fig. 6A). Modules 13, 15, 17 and 25 had GSEA enrichment scores (ES) of 0.37, 0.44, 0.58 and 0.32, respectively (P < 1.0 × 10^−4^) (Fig. 6B–E). The expression of genes in all four modules was decreased in *Snca*^−/−^ mice (Fig. 6F–I). Though the alterations in expression were subtle (~4–8%), they were consistent across nearly all module genes (Fig. 6F–I).

If the Kme values generated in the L5 network are reflective of the real underlying bone network structure, then we would anticipate that the expression of more highly connected genes (higher Kme) in each module would be more severely perturbed by *Snca* deficiency. Indeed in all four modules, higher gene connectivity was correlated (module 13 = r_s_ = 0.38, P = 5.1 × 10^−12^; module 15 = r = 0.31, P = 1.3 × 10^−7^; module 17 = r_s_ = 0.54, P < 2.2 × 10^−16^ and module 25 = r_s_ = 0.57, P < 2.2 × 10^−16^) with more significant (−log10(Pvalue)) differences in expression between *Snca* genotypes (Fig. 6J–M).

These four modules were also located on the same branch of the eigengene network (Fig. 1) and they were enriched in genes belonging to similar GO categories. Modules 13 and 25, whose eigengenes clustered together, were enriched for GO terms such as “contractile fiber”, “myofibril” and “cytoskeleton” (Supplemental Table 4). In contrast, modules 15 and 17 were enriched for genes belonging to terms such as “mitochondrion” and “oxidative phosphorylation” (Supplemental Table 4). Genes in all four modules were highly expressed in skeletal muscle, though they demonstrated relatively high expression in primary calvarial osteoblast cultures especially early in differentiation (day 5 and 14) (Supplemental Fig. 1).

To confirm that *Snca* was expressed in purified osteoblasts and might be altering the expression of the four modules in osteoblasts, we used RNA-seq data (GSE54461) from FACs sorted primary calvarial cells expressing Cyan Fluorescent protein (CFP) driven by a Col3.6 promoter^29^. These data revealed that *Snca* expression increased early in differentiation and decreased in the later stages of osteoblastogenesis (Supplemental Fig. 1).

**Figure 7:**
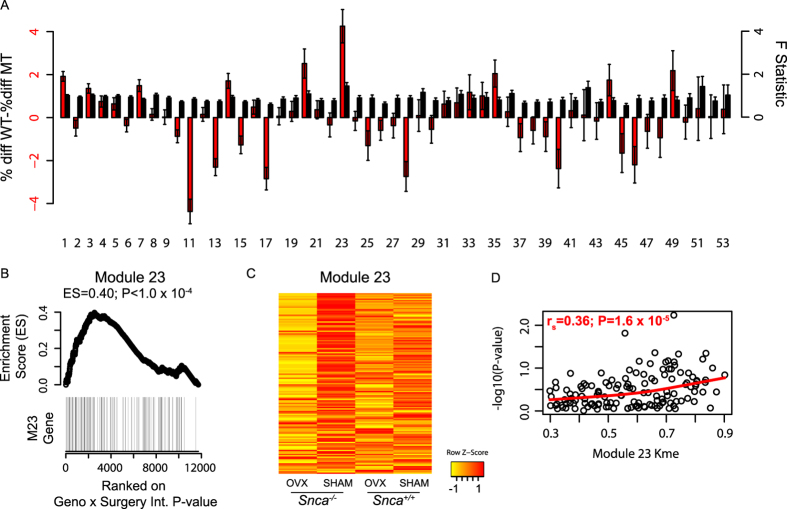
*Snca* deficiency specifically alters the expression of genes in its own module 23. (**A**) The percent difference in module 23 genes was higher in *Snca*^−/−^ mice. The average F-statistic for the genotype x surgery ANOVA term was highest in module 23. (**B**) Gene Set Enrichment Analysis (GSEA) results for module 23. The differences in expression between OVX and SHAM within genotype were ranked from most significant to least significant. Module 23 genes tend to be among the most significant. (**C**) Module 23 genes were differentially expressed as a function of OVX, but only in *Snca*^−/−^ mice. (**F**) In module 23 we observed a positive correlation between Kme and differential expression (−log10(Pvalue)). Values in A are means ± s.e.m.

As predicted based on its high connectivity, the expression of module 23 genes was altered as a function of *Snca* genotype. However, the modulation was not strictly a result of *Snca* genotype, but rather of a genotype x surgery interaction (Fig. 7A). Specifically, the difference in expression of module 23 genes between OVX and SHAM was greater in *Snca*^−/−^ compared to *Snca*^+/+^ mice (Fig. 7A). Module 23 was the only module with a significant difference in behavior based on GSEA (ES = 0.40, P < 1.0 × 10^−4^) (Fig. 7B). Module 23 genes were the lowest in *Snca*^−/−^ OVX mice, whereas they were the highest in *Snca*^−/−^ SHAM mice (Fig. 7C). Additionally, more highly connected genes had more significant genotype x surgery interactions (Fig. 7D). Taken together these results indicate that *Snca* specifically regulates, either directly or indirectly, bone network modules 13, 15 17, 25 and its own, 23.

## Discussion

In the U.S., 80% of the total osteoporotic population is comprised of postmenopausal women, 50% of which will experience a fracture during their lifetime. The high prevalence of osteoporosis and fractures in women is primarily due to postmenopausal bone loss. In a mouse model of postmenopausal bone loss, we used network analysis to identify genes influencing the skeletal response to estrogen deficiency. Through this approach, we identified a co-expression module whose overall expression levels were decreased by OVX. This module was enriched in genes involved in the response to oxidative stress and its homologs were co-expressed in human bone marrow. *Snca* was one of the most connected genes in module 23. We demonstrated that its deficiency provided partial protection from bone loss after OVX and the attenuation was associated with the specific alteration of five network modules, including its own module 23. These data identify module 23 as a network of genes involved in the skeletal response to OVX and implicate *Snca* as a regulator of bone network homeostasis and OVX-induced bone loss.

Our goal was to identify novel genes influencing bone loss due to estrogen depletion. As a starting point, we generated expression profiles of L5 vertebrae from three inbred strains of mice and identified 153 differentially expressed genes in bone as a function OVX. However, based on these data alone it was difficult to know which genes might be key drivers of OVX-induced bone loss. Co-expression network analysis allowed us to begin to address this limitation by identifying gene modules whose expression was decreased by OVX. In modules associated with a phenotype, connectivity has been shown to be a strong, biologically motivated, measure of a gene’s importance in the regulation of the phenotype^30^. Therefore, by switching the focus from individual gene expression differences to network-level differences in module behavior, we were able to more effectively dissect a key component of the skeletal response to OVX.

*SNCA* is a 140 amino acid protein whose function is unclear^31^. Much of what is known regarding SNCA revolves around its central role in the etiology of Parkinson’s Disease (PD)^31^. Misfolded aggregates of SNCA are the predominant component of Lewy Bodies, the primary pathological agent in PD^32^. Specific point mutations in *SNCA* have been associated with familial, monogenic forms of PD^25^. Additionally, common variants in the *SNCA* gene have recently been identified as associated with complex forms of PD^27^,^33^. Considerable evidence suggests that point mutations and increased expression of *SNCA* result in its aggregation, which promotes mitochondrial dysfunction and oxidative stress^31^,^34^. Interestingly, there is a high of prevalence of osteoporosis in PD which cannot be fully ascribed to the increased risk of falling and postural instability seen in PD^35^,^36^. Indeed low BMD alone is strongly associated with PD independent of muscle tone or concomitant medications^36^. Studies outside the central nervous system have revealed that *SNCA* is highly expressed in erythroblasts and its protein levels remain high in mature erythrocytes in both bone marrow and circulation^37^,^38^. Furthermore, erythrocytes from *Snca*^−/−^ bone marrow have lower levels of ROS and oxidative stress^24^. Increased oxidative stress has been shown to promote OVX-induced bone loss^23^. These data are consistent with the observation that the absence of *Snca* resulted in a reduction in OVX-induced bone loss.

In *Snca*^−/−^ mice bone loss after OVX was attenuated and the expression of genes in modules 13, 15, 17 and 25 were lower. The alterations were subtle, but significant and consistent, especially for the most highly interconnected genes in these modules. This observation provides independent validation that genes in all four modules are co-expressed, as well as insight into the mechanism through which *Snca* affects OVX-induced bone loss. Although, definitive proof will require further experimentation, it is possible that perturbation of one or more modules is responsible for *Snca* mediated protection from OVX-induced bone loss. These data also suggest, as a module hub, that *Snca* directs the expression of these four modules either directly or indirectly. There is no evidence, to our knowledge, that *Snca* is capable of directly regulating transcription or transcript stability. However, it does physically interact with a large number of proteins, some of which are directly involved in transcriptional regulation (http://thebiogrid.org/112506/summary/homo-sapiens/snca.html), suggesting that the effect of *Snca* on module gene expression is through indirect mechanisms.

Genes in modules 13, 15, 17 and 23 were decreased in *Snca*^−/−^ mice, independent of surgical status. These modules contain several genes that are highly expressed in skeletal muscle. Though care was taken to remove skeletal muscle tissue from dissected vertebras, it is probable that contaminating muscle tissue was a source for part of the expression of genes in these modules. However, *Snca* is not expressed in skeletal muscle, nor is it likely that it would cause differential contamination. Intriguingly, *Snca* and many genes in modules 13, 15, 17 and 23 are expressed in osteoblasts, particularly early in differentiation (Supplemental Fig. 1). Additionally, Alam *et al*. found that *Snca* expression in osteoblasts correlated with BMD in congenic P/NP rats^39^. Based on these data, the decrease in the expression of these four modules may be osteoblast specific. Further work will be required to determine the effect that *Snca* deficiency has, if any, on osteoblast activity and subsequently OVX-induced bone loss.

Module 23 contained 131 genes, and as described above, many are known to be involved in the response to oxidative stress, including *Snca*, *Prdx2, Prdx3 and Gpx1*. A common characteristic of the remaining genes is high expression in bone marrow. Due to their primary role in oxygen transport, bone marrow erythocytes generate high levels of reactive oxygen species (ROS)^40^. In bone marrow, oxidative stress activates T-cells leading to increased production of tumor necrosis factor α (*Tnfα*). *Tnfα* increases the levels, and the responsiveness of osteoclasts, to osteoclastogenic molecules such as receptor activator of nuclear factor kappa-B ligand (RANKL), macrophage colony- stimulating factor (M-CSF) and Interleukins-1 (IL1), -6 (IL6) and -7 (IL7), resulting in enhanced osteoclast bone resorption and increased bone loss^23^. In addition to oxidative stress, many of the module 23 genes are involved in erythrocyte function and differentiation (e.g. *Uros*^41^, *Tgfbr3*^42^ and *Dyrk3*^43^). *Uros* was the most highly connected gene in module 23. These data suggest that module 23 controls the production of ROS in bone marrow either through intracellular mechanisms (e.g. modifying the redox status of erythrocytes), by influencing the number or function of erythrocytes or both.

The expression of module 23 genes differed to a larger extent between OVX and SHAM in *Snca*^−/−^, as compared to wild-type littermates. This was predominantly due to an increase in module 23 gene expression in *Snca*^−/−^ SHAM mice. Given the previous report demonstrating lower oxidative stress in *Snca*^−/−^24, we predict that the increase in module 23 gene expression is present at the time of surgery. This increase would boost the antioxidant capacity of bone marrow in *Snca*^−/−^ mice and provide protection against OVX-induced bone loss.

This study does have limitations. First, the networks were generated using a relatively small panel of inbred strains. We would expect a larger number of strains would lead to a more informative network. Importantly, studying a larger number of strains would also allow the discovery of modules whose behavior correlate with strain differences in the response to OVX. Sixteen-week old C57BL/6J females loss little vertebral trabecular bone (BV/TV) four weeks post-OVX (Table 1); however, the *Snca* knockout mice we used in this experiment on a C57BL/6J background. In C57BL/6J females, vertebral BV/TV peaks at 8 weeks of age and then decreases throughout life^44^. The loss of bone is most rapid between 8 and 16 weeks^44^. Bone loss after OVX is more pronounced in C57BL/6J mice, when mice are ovariectomized at 8 weeks^45^. Therefore, in order to determine if *Snca* was involved in OVX-induced bone loss, we performed surgeries in 8-week-old *Snca* mutant and wild-type females instead of 16-week-old females. Given the age differences between the mice used to generate the co-expression networks and the *Snca* mutants, it is possible the affect of *Snca* deficiency impact different processes than those occurring in older mice. Future work looking at the effects of *Snca* deficiency on different strains on mice (such as BALB/cJ) will be performed to clarify the precise role of Snca in OVX-induced bone loss in 16-week old mice.

One of the motivations for this work is the lack of unbiased genome-wide studies of postmenopausal bone loss in humans. Even though postmenopausal bone loss is heritable (H2 = 40 to 60%)^46^,^47^,^48^,^49^,^50^,^51^, it has not been subjected to genomic or genetic analyses, such as genome-wide association studies (GWASs). This is primarily due to the fact that human bone loss is a challenging phenotype because it requires longitudinal BMD measurements taken many years apart and bone loss determined by clinical scans is associated with a high level of measurement noise^52^,^53^. Also, individual rates of loss vary as a function of time after menopause, leading to an additional source of variation^52^,^53^. Together, these challenges suggest that large-scale GWAS for bone loss in the future will be very challenging, if not impossible. As a result we believe that unbiased genomic and genetic approaches in the mouse, as we describe here, are critical to unravel the molecular and genetic basis of bone loss due to estrogen deficiency, which represents the single most important clinical problem in the osteoporosis field. We are planning studies in larger numbers of mouse strains that will allow us to identify genetic factors that drive differences in OVX-induced bone loss.

In summary, we have applied network analysis to bone expression profiles from multiple inbred strains of mice to identify a module of genes whose expression is decreased as a result of OVX. By mining this module, we implicate *Snca* as a novel regulator of OVX-induced bone loss. Our data suggest that *Snca* deficiency attenuates bone loss in part by regulating the oxidative environment of bone marrow through the expression of genes in specific bone network modules. This work increases our molecular understanding of the response of bone to estrogen deficiency and has implications for developing approaches to combat postmenopausal bone loss. It also demonstrates the utility of co-expression network analysis for gene discovery.

## Methods

### Animal Procedures

Female BALBC/J (Stock #000651), C3H/HeJ (Stock #000659), C57BL6/J (Stock #000664) and *Snca*^*tm1Rosl*^/J (Stock #003692) mice were purchased from the Jackson labs. At 16 weeks of age females from the three classical inbred strains were randomly assigned to SHAM or OVX surgical groups (N = 8/group/strain). Males homozygous for the *Snca*^*tm1Rosl*^ allele (*Snca*^−/−^) were bred to C57BL6/J females and *Snca*^*+/−*^ mice were intercrossed generating *Snca*^+/+^ and *Snca*^−/−^ littermate females. At 8 weeks of age *Snca*^+/+^ and *Snca*^−/−^ females were randomly assigned to SHAM or OVX surgical groups (N = 6–10 biological replicates per genotype and surgical group). Mice were anesthetized with isoflurane. Using sterile technique, surgery was performed using a dorsal approach. Small lateral incisions were made in the abdominal wall to exteriorize and excise the ovaries, and the abdominal and skin incisions were subsequently closed. Analgesia was provided with administration of Bupivacaine and Ketoprofen. Before and after surgery, mice were given free access to food and water. All mice were euthanized four weeks post-surgery. The success of OVX surgeries was confirmed by measuring uterine weights^19^. In all experiments uterine weights were on average four-fold lower in OVX animals with non-overlapping distributions. L4 vertebras were collected and fixed in 70% EtOH for μCT analysis. The L5 vertebras were homogenized in Trizol for subsequent RNA isolation. The individuals performing the μCT and microarray analysis were blinded to experimental group. Target sample sizes (N = 8 per experimental group) were determined, based on previous experimental results, to provide 80% power to detect difference in most phenotypes analyzed. The study was carried out in strict accordance with NIH’s Guide for the Care and Use of Laboratory Animals. Additionally, the University of Virginia Institutional Animal Care and Use Committee approved all animal procedures.

### μCT analysis

Microarchitecture of L4 vertebras were quantified post-mortem by high-resolution micro-computed tomography (MicroCT40, Scanco Medical AG, Switzerland). Approximately 100 CT slices with an isotropic voxel size of 12 μm were taken just proximal to the distal growth plate for trabecular bone measurements.

### RNA and microarray processing

Dissected L5 vertebras were immediately homogenized in Trizol and lysates were frozen at −80 °C until RNA isolation. Total RNA was isolated using the Trizol Plus RNA Purification Kit (Invitrogen, Carlsbad, CA). RNA integrity was confirmed using a 1% agarose gel (Agilent, Palo Alto, CA). Microarray expression profiles were generated using the Illumina MouseWG-6 v2 BeadChips (Illumina, San Diego, CA) in the Genome Science Laboratory at UVa. Biotin-labeled cRNA was synthesized using the total prep RNA amplification kit from Ambion (Austin, TX). cRNA was quantified and normalized to 77 ng/μl, and then 850 ng was hybridized to Beadchips. Raw expression values were quantile normalized using the affy R package^54^ and log_2_ transformed. Data QC identified one outlier array in the strain survey from the BALB/cJ SHAM group. Data from this sample was not included in any downstream analysis. The array data from both the strain survey and *Snca* mutant analysis are available from GEO (GSE68313).

### Differential expression analysis

A standard differential expression analysis was used to identify probes with expression differences as a function of strain and OVX. Probes with an Illumina detection P < 0.01 in 50% of samples belonging to any strain and/or surgery group were retained. A linear model including strain and surgery was fitted to normalized data: 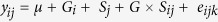, where *y*_*ij*_ is the normalized-transformed gene expression, *μ* is the population mean, *G*_*i*_ is the effect of *i*^*th*^ strain genotype, *S*_*j*_ is the effect of *j*^*th*^ surgery, *G* × *S*_*ij*_ is the effect of strain genotype by surgery interaction, and *e*_*ijk*_ is the residual effect. Using ANOVA in the R/Maanova R package^55^ we tested the significance of effects from strain genotype, surgery and their interaction. Empirical P-values were calculated by performing 1000 permutations and then corrected for multiple comparisons using a 10% false-discovery rate (FDR) cutoff^56^.

### Transcriptional Network Analysis

Network analysis was performed using the WGCNA R package^10^,^57^,^58^. To generate co-expression networks, we first calculated Pearson correlation coefficients for all gene-gene comparisons across all microarray samples for probes with an Illumina detection P < 0.01. The matrix of correlations was then converted to an adjacency matrix of connection strengths. The adjacencies were defined as 
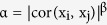
, where x_i_ and 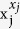 are the i^th^ and j^th^ gene expression traits. The power β was selected using the scale-free topology criterion previously outlined by Zhang and Horvath^12^. In this study, we used a β = 8. Modules were defined as sets of genes with high topological overlap^59^. The topological overlap measure (TOM) between the i^th^ and j^th^ gene expression traits was taken as 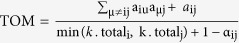, where 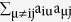 denotes the number of nodes to which both i and j are connected, and μ indexes the nodes of the network. A principal component analysis was used to generate a vector of values (first principal component) that summarized a modules behavior. Intramodular connectivity (kme) was defined as the correlation between a gene’s expression and its module eigengene. Network depictions were constructed using Cytoscape^60^. Gene Ontology enrichment analyses were performed using the RDAVIDWebService R package^61^.

### Human bone marrow network

To determine if module 23 genes were also co-expressed in human bone marrow we used publically available microarray data on bone marrow from human donors available from GEO (GSE11504). These data were from 25 healthy donors^62^. We used these data to create a co-expression network using the same parameters (β = 8) as used in the mouse co-expression network outlined above. This network was used to compare module 23 genes and their human homologs as described in results.

### Gene Set Enrichment Analysis (GSEA)

To assess the significance of coordinated differences in the *Snca* perturbation experiment we used Gene Set Enrichment Analysis (GSEA)^28^. Analyses were performed using the GSEA R script (http://www.broadinstitute.org/cancer/software/gsea/wiki/index.php/R-GSEA_Readme). GSEA was designed to assess the significance of subtle, coordinated changes in genes belonging to a similar functional unit (i.e. pathway). GSEA takes a ranked list of values (the three metrics described above) and calculates an “enrichment score” (ES) of genes belonging to a grouping (in our case module) using a running-sum statistic that corresponds to a weighted Kolmogorov–Smirnov-like statistic^28^. Permutations (1000) were used to assess the significance of enrichment scores.

### Additional statistical analyses

All statistical methods were employed using the R software environment for statistical computing and graphics^63^. The effects of strain genotype, surgery and their interactions on bone mass traits were tested using the following linear model: 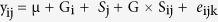, where y_ij_ is the normalized-transformed gene expression, μ is the population mean, G_i_ is the effect of i^th^ strain genotype, S_j_ is the effect of j^th^ surgery, G × S_ij_ is the effect of strain genotype by surgery interaction, and e_ijk_ is the residual effect. The significance of effects from strain genotype, surgery and their interaction was tested using ANOVA. Differences in bone traits after OVX within strain were determined using a Tukey’s multiple comparison test. Percent change in bone mass phenotypes in *Snca*^+/+^ and *Snca*^−/−^ mice were calculated as the difference between OVX and SHAM groups taken as a percent of SHAM. The significance of percent change measures was determined using ANOVA. Significance for all above tests were set at P < 0.05.

## Additional Information

**How to cite this article**: Calabrese, G. *et al*. Network Analysis Implicates Alpha-Synuclein (*Snca*) in the Regulation of Ovariectomy-Induced Bone Loss. *Sci. Rep.* **6**, 29475; doi: 10.1038/srep29475 (2016).

## Supplementary Material

Supplementary Information

Supplementary Table S1

Supplementary Table S2

Supplementary Table S3

Supplementary Table S4

## Figures and Tables

**Table 1:** Effects of OVX on vertebral bone traits across three inbred strains.

	BALBc/J			C3H/HeJ			C57BL6/J			P-values		
	SHAM (N = 8)	OVX (N = 8)	P	SHAM (N = 7)	OVX (N = 8)	P	SHAM (N = 8)	OVX (N = 8)	P	Genotype	Surgery	G × S
BV/TV (%)	27.5 ± 1.0	22.9 ± 0.7	**6.8 × 10**^**−3**^	13.4 ± 0.7	12.0 ± 0.4	0.86	22.6 ± 1.1	21.5 ± 1.0	0.95	**<*****2.2***** × *****10***^**−16**^	**1.8 × 10**^**−3**^	0.10
Tb.N (1/mm)	3.6 ± 0.1	3.3 ± 0.1	0.39	2.4 ± 0.1	2.3 ± 0.1	0.99	3.9 ± 0.2	4.1 ± 0.1	0.81	**<*****2.2***** × *****10***^**−16**^	0.55	0.09
Tb.Th (μm)	62.0 ± 1.4	56.3 ± 0.5	**3.0 × 10**^**−3**^	59.9 ± 1.6	55.6 ± 0.9	0.06	55.3 ± 0.8	51.2 ± 0.7	0.07	***2.1***** × *****10***^**−*****6***^	**1.5 × 10**^**−6**^	0.69
Tb.Sp (μm)	301.4 ± 8.6	330.3 ± 10.2	0.51	441.6 ± 18.0	450.8 ± 14.3	0.99	268.6 ± 10.6	247.3 ± 8.5	0.79	**<*****2.2***** × *****10***^**−16**^	0.57	0.11

**Table 2:** Bone co-expression network modules significantly associated (P < 9.4 × 10^−4^) with strain genotype and/or surgery.

Module	No. Probes	P-Geno	P-Surgery	P-Int
1	1124	1.0 × 10^−11^	1.9 × 10^−3^	0.99
2	815	1.9 × 10^−19^	2.7 × 10^−3^	0.20
4	668	4.3 × 10^−17^	0.12	0.53
5	647	3.3 × 10^−11^	0.85	0.72
6	645	2.0 × 10^−22^	0.58	0.33
9	414	1.7 × 10^−15^	0.11	0.48
11	382	0.97	3.7 × 10^−6^	0.10
20	140	0.01	1.7 × 10^−6^	0.69
23	136	0.32	1.5 × 10^−5^	0.95
42	56	1.7 × 10^−4^	9.3 × 10^−3^	0.19
51	34	3.3 × 10^−7^	1.4 × 10^−5^	0.67
