# Supplementary material for: Network Analysis Implicates Alpha-Synuclein (Snca) in the Regulation of Ovariectomy-Induced Bone Loss
Source: Sci Rep. 2016 Jul 5;6:29475. doi: 10.1038/srep29475 (PMC4932518; doi:10.1038/srep29475)

Supplementary Information for:

Network Analysis Implicates Alpha-Synuclein (*Snca*) in the Regulation of Ovariectomy-Induced Bone Loss

Gina Calabrese[1], Larry D. Mesner[1], Patricia L. Foley[2], Clifford J. Rosen[3] and Charles R. Farber[1,4]

[1]Center for Public Health Genomics, University of Virginia, Charlottesville, Virginia 22908, USA
[2]Department of Microbiology and Immunology, Georgetown University, Washington, DC 20007, USA
[3]Maine Medical Center Research Institute, 81 Research Drive, Scarborough, Maine 04074, USA
[4]Departments of Public Health Science and Biochemistry and Molecular Genetics, University of Virginia, Charlottesville, Virginia 22908, USA

Running Title: *Snca* and bone loss

Correspondence should be addressed to:
Charles R. Farber
Center for Public Health Genomics
P.O. Box 800717
University of Virginia
Charlottesville, VA 22908
Tel: 434-243-8584
Fax: 434-982-1815
Email: crf2s@virginia.edu

Supplemental Figure 1

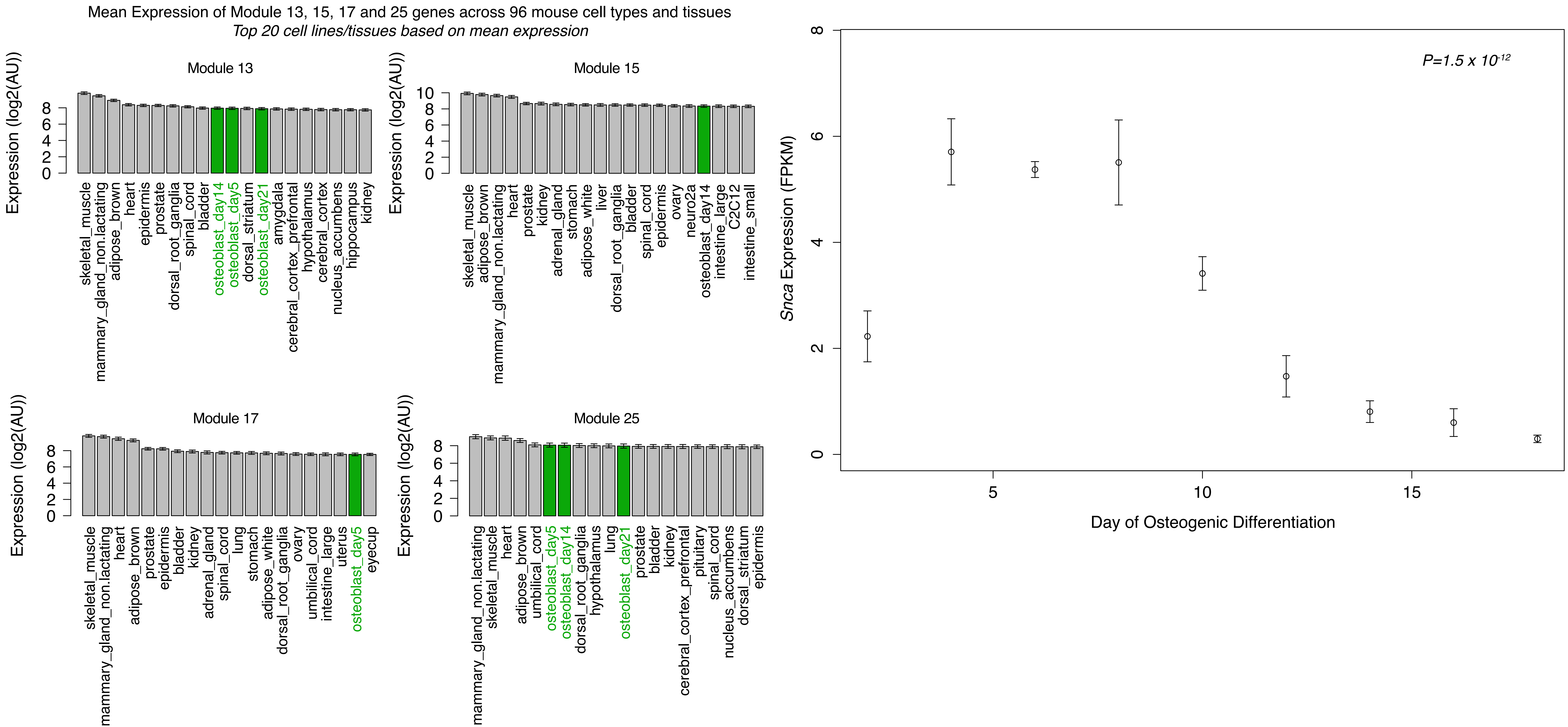

Supplement: Supplementary Information [file srep29475-s1.pdf]
